# Development of RT-PCR Assays for Simple Detection and Identification of Sabin Virus Contaminants in the Novel Oral Poliovirus Vaccines

**DOI:** 10.3390/vaccines13010075

**Published:** 2025-01-15

**Authors:** Olga Singh, Hasmik Manukyan, Erman Tritama, Shwu-Maan Lee, Jerry P. Weir, Majid Laassri

**Affiliations:** 1 Center for Biologics Evaluation and Research, US Food and Drug Administration, Silver Spring, MD 20993, USA; olga.singh@fda.hhs.gov (O.S.); hasmik.manukyan@nih.gov (H.M.); jerry.weir@fda.hhs.gov (J.P.W.); 2 Research and Development Division, PT. BioFarma, Bandung, West Java 40161, Indonesia; erman.tritama@biofarma.co.id; 3 Center for Vaccine Innovation and Access, PATH, Seattle, WA 98121, USA; smlee@path.org

**Keywords:** viral contamination, OPV, nOPV, RT-PCR, vaccines, poliovirus, viral detection

## Abstract

Background/Objectives: Conventional live oral poliovirus vaccines (OPVs) effectively prevent poliomyelitis. These vaccines are derived from three attenuated Sabin strains of poliovirus, which can revert within the first week of replication to a neurovirulent phenotype, leading to sporadic cases of vaccine-associated paralytic poliomyelitis (VAPP) among vaccinees and their contacts. A novel OPV2 vaccine (nOPV2) with enhanced genetic stability was developed recently; type 1 and type 3 nOPV strains were engineered using the nOPV2 genome as a backbone by replacing the capsid precursor polyprotein (P1) with that of Sabin strains type 1 and type 3, respectively. The nOPV vaccines have a high degree of sequence homology with the parental Sabin 2 genome, and some manufacturing facilities produce and store both Sabin OPV and nOPV. Therefore, detecting Sabin virus contaminations in nOPV lots is crucial. Methods: This study describes the development of pan quantitative reverse transcription polymerase chain reaction (panRT-PCR) and multiplex one-step RT-PCR (mosRT-PCR) assays for the straightforward detection and identification of contaminating Sabin viruses when present in significantly higher amounts of nOPV strains. Results: The two assays exhibit high specificity, reproducibility, and sensitivity to detect 0.0001% and 0.00001% of Sabin viruses in nOPV, respectively. Additionally, an analysis of 12 trivalent nOPV formulation lots using both methods confirmed that the nOPV lots were free from Sabin virus contamination. Conclusions: The results demonstrated that the RT-PCR assays are sensitive and specific. These assays are relevant for quality control and lot release of nOPV vaccines.

## 1. Introduction

Poliomyelitis (polio) is a highly infectious disease caused by three poliovirus serotypes. The virus spreads primarily via the fecal-oral route and, less frequently, through contaminated food or water. Two types of vaccines are available to protect against polio: the Trivalent Inactivated Poliovirus Vaccine (IPV) and the Trivalent Sabin Live Oral Poliovirus Vaccine (tOPV). The tOPV is composed of three genetically unstable Sabin strains that can revert swiftly to neurovirulence during replication in vaccine recipients and infected persons, occasionally resulting in vaccine-associated paralytic polio cases and in circulating vaccine-derived polioviruses (cVDPVs) [1,2].

The Global Polio Eradication Initiative has significantly reduced polio cases worldwide, successfully eradicating wild poliovirus types 2 and 3 in 2015 and 2019, respectively [3,4]. Currently, only wild poliovirus type 1 is still circulating in certain regions of Afghanistan and Pakistan. Since 2016, cVDPV2 has been the predominant cause of poliovirus outbreaks. To mitigate the risk of cVDPV2, vaccination with trivalent Sabin OPV was replaced in 2016 by bivalent OPV (bOPV), which includes Sabin strains 1 and 3, co-administered with IPV [5,6].

However, IPV is known to induce insufficient mucosal immunity to prevent poliovirus circulation and transmission [7,8,9]. Consequently, recipients of bOPV and IPV remain vulnerable to type 2 poliovirus infection and subsequent spread of the virus [10,11,12]. Following the switch in immunization strategy, cVDPV2 outbreaks rose globally [13]. To control these outbreaks, monovalent OPV2, derived solely from the Sabin 2 virus, was instituted, leading to further cVDPV2 outbreaks [14,15].

Recently, a novel OPV2 (nOPV2) vaccine was developed [16] by optimizing the Sabin 2 genome to reduce the chance of reversion to a neurovirulent phenotype [16]. nOPV1 and nOPV3 were created from the nOPV2 genome by replacing its capsid precursor polyprotein (P1) with the P1 of Sabin strains type 1 and 3, respectively [17]. BioFarma (Indonesia, Bandung) is developing trivalent nOPV (tnOPV) as a product [18].

As per the polio eradication strategy, the WHO recommends introducing nOPV 1 and 3 vaccines in cVDPV epidemic regions [19]. nOPV1 and nOPV3 vaccines are anticipated to be available through the WHO prequalification process in the near future [19].

Since tnOPV is derived from Sabin viruses and vaccine manufacturers may store both nOPV and Sabin OPV vaccines at the same facility, preventing contamination of nOPV lots with Sabin viruses through careful manufacturing controls and quality testing is crucial.

Multiple molecular methods for detecting Sabin strains have been developed, including quantitative reverse transcription polymerase chain reaction (RT-PCR) [20] and quantitative multiplex one-step RT-PCR (qmosRT-PCR), which detect and identify the three Sabin OPV strains [21]. However, none of these methods was specifically designed to distinguish Sabin strains from their nOPV counterparts. We developed an RT-PCR assay to detect the Sabin 2 virus in high quantities of nOPV2 virus [22].

In this report, we propose a simple panRT-PCR assay for universal detection of Sabin strains in nOPV vaccines, alongside a mosRT-PCR assay for specific detection and identification of each Sabin strain in nOPV vaccines using tailored primers and TaqMan probes. The assays are simple, specific, and sensitive, making them suitable for quality control and lot release of nOPV vaccines.

## 2. Materials and Methods

### 2.1. Vaccine Viruses and nOPV Batches

The OPV Sabin strains (GenBank accession numbers: AY184219 for Sabin 1, AY184220 for Sabin 2, and AY184221 for Sabin 3) served as positive controls for each run of quantitative PCR assays and for preparing spiking samples. BioFarma provided nOPV types 1, 2, and 3, along with bulks for 12 trivalent nOPV formulation lots. We prepared spiking samples by adding Sabin viruses into nOPV viruses and performed RNA extraction in a Biosafety level (BSL) 3 laboratory, followed by downstream analysis in a BSL2 laboratory.

### 2.2. Primers and TaqMan Oligoprobes

**Figure 1:**
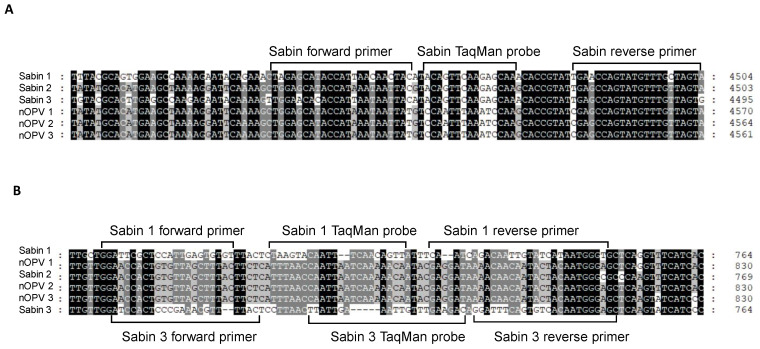
Genomic location of primers and TaqMan probes used for detection and identification of Sabin viruses in nOPV vaccines: (**A**) primers and TaqMan probe used in panRT-PCR assay; (**B**) primers and TaqMan probes used in mosRT-PCR assay for detection and Sabin 1 and 3 strains.

For the panRT-PCR assay, we designed primers and a TaqMan probe for universal detection of Sabin viruses in nOPV, primarily based on the TaqMan probe’s ability to distinguish between Sabin and nOPV strains (Figure 1A). Amplification with this primer set yielded a DNA amplicon of 70 base pairs (Figure 1A and Table 1, rows 3–5). For the mosRT-PCR assay, unique primers and the TaqMan probe for detecting and identifying Sabin 2 virus in nOPV have been described previously (Table 1, rows 11–13) [22]. Primers and probes for detecting and identifying Sabin 1 and 3 viruses are shown in Figure 1B and Table 1 (rows 7–9 for Sabin 1; rows 15–17 for Sabin 3).

Amplification with mosRT-PCR primers resulted in DNA amplicons of 79, 68, and 75 base pairs for Sabin 1, 2, and 3, respectively. Primers were sourced from Integrated DNA Technologies, Inc. (Coralville, IA, USA), and TaqMan oligoprobes from Thermo Fisher Scientific (South San Francisco, CA, USA).

Amplification with mosRT-PCR primers resulted in DNA amplicons of 79, 68, and 75 base pairs for Sabin 1, 2, and 3, respectively. Primers were sourced from Integrated DNA Technologies, Inc. (Coralville, IA, USA) and TaqMan oligoprobes from Thermo Fisher Scientific (San Francisco, CA, USA).

### 2.3. Extraction of Viral RNA

We isolated total RNA from nOPV, Sabin strains, and spiked samples using the QIAamp Viral RNA Mini Kit (QIAGEN, Chatsworth, CA, USA) per the manufacturer’s instructions. The isolated RNA was eluted in water treated with Diethyl Pyrocarbonate (DEPC) and stored at −80 °C.

### 2.4. mosRT-PCR Amplification

We prepared mosRT-PCR reactions in micro-96-well plates (Thermo Fisher Scientific) in a final volume of 20 μL, using 2 μL of viral RNA and the QuantiNova Multiplex RT-PCR Kit (QIAGEN, Valencia, CA, USA). TaqMan probes Sab1PrbVIC, Sab2PrbFAM, and Sab3PrbNED for Sabin 1, 2, and 3 viruses, respectively, were used at a final concentration of 25 nM each, alongside their corresponding three pairs of primers at a concentration of 0.8 μM each (Table 1). We conducted the mosRT-PCR run using the ViiA7 Real-Time PCR System (Thermo Fisher Scientific) under the following conditions: one cycle at 50 °C for 10 min, followed by 2 min at 95 °C, and 45 cycles of 5 s at 95 °C, 10 s at 50 °C, and 30 s at 60 °C.

### 2.5. panRT-PCR Amplification

Similarly to the mosRT-PCR reactions, we prepared panRT-PCR reactions in micro-96-well plates with a final volume of 20 μL, using 2 μL of viral RNA and the QuantiNova Multiplex RT-PCR Kit (QIAGEN, Valencia, CA, USA). We employed the SabUprbFAM Universal TaqMan probe for Sabin 1, 2, and 3 viruses at a final concentration of 50 nM, with its corresponding pair of primers at a concentration of 2.4 μM each (Table 1). The panRT-PCR run was performed on the ViiA7 Real-Time PCR System under conditions identical to those of the mosRT-PCR reactions.

### 2.6. Spiking Sample Preparation for Sensitivity Evaluation of the Assays

We spiked Sabin strains 1, 2, and 3 individually and in mixtures into tnOPV, which contained 6.85, 6.25, and 6.47 Log_10_ CCID_50_/mL of nOPV 1, 2, and 3, respectively. Percentages of Sabin viruses in tnOPV were calculated based on virus titer expressed in CCID_50_/mL and Genome Copy Number (GC#/mL), as shown in Table 2. Virus titers were determined using a conventional CCID_50_ assay [23] and the MPBT assay [18]. We calculated the GC# of the viruses as described previously [24]. Spiked samples underwent RNA extraction as previously outlined, and we used the resulting RNA samples for sensitivity evaluation of panRT-PCR and mosRT-PCR assays.

## 3. Results

### 3.1. Design of Specific Primers and Probes for Sabin Virus Detection in nOPV

For the panRT-PCR assay, we designed the probe and primers within the 2C protein region (4459–4503 nt in the Sabin 1 genome). The probe specifically distinguished all three Sabin strains from nOPV viruses, while the primers amplified both Sabin and nOPV viruses, with slightly reduced specificity for nOPV viruses (Table 1). Thus, the panRT-PCR assay effectively detected all three Sabin viruses while distinguishing them from nOPV strains based on the probe’s discriminating characteristic.

For the mosRT-PCR assay, we designed primers and probes within the 5′ UTR region. Specific primers and probe for the Sabin 1 strain are located in the 670–748 nt region of the Sabin 1 genome, while those for the Sabin 3 strain are located in the 675–749 nt region of the Sabin 3 genome. Other primers and probe for detecting the Sabin 2 strain in nOPV [22], located in the 477–544 nt region of the IRES element in the 5′ UTR of the Sabin 2 genome, were also utilized. The mosRT-PCR assay’s primers and probes were carefully designed for the specific detection and identification of each Sabin strain in nOPV (Table 1).

### 3.2. Evaluation of Assay Specificity

**Figure 2:**
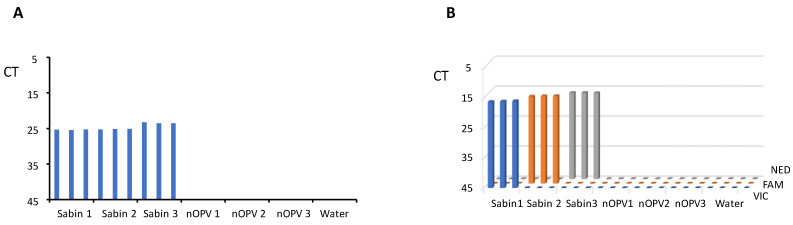
Evaluation of the assays’ specificity. (**A**) panRT-PCR assay; (**B**) mosRT-PCR assay.

In the same run of the panRT-PCR assay, we tested RNA samples from each of Sabin strains 1, 2, and 3, nOPV strains 1, 2, and 3, and water (negative control) in triplicate. The results (Figure 2A) demonstrated that the panRT-PCR could detect all three Sabin strains but no nOPV viruses, confirming the assay’s specificity for Sabin strains.

Similarly, we employed mosRT-PCR to analyze RNA samples of Sabin and nOPV strains, as well as water, in triplicate. Results (Figure 2B) indicated that the mosRT-PCR assay successfully detected and identified each Sabin type using its specific dye (VIC for Sabin 1, FAM for Sabin 2, and NED for Sabin 3), with no observed interference among dyes and no nOPV viruses. This confirmed the mosRT-PCR assay’s specificity for detecting and identifying each Sabin strain.

### 3.3. Sensitivity and Linearity

To assess the sensitivity and linearity of both assays, we spiked Sabin 1, 2, and 3 strains individually and in mixtures into tnOPV containing 6.85, 6.25, and 6.47 Log_10_ CCID_50_/mL of nOPV 1, 2, and 3 strains, respectively. We calculated the percentage of Sabin viruses using the formula: % (X) = ([X]/[X + A+B + C]) x 100, where (X) is, for example, the titer of Sabin 1 (where only Sabin 1 spiked in tnOPV), and A, B, and C are the titers of nOPV1, 2, and 3, respectively. The prepared spiked samples are shown in Table 2.

#### 3.3.1. panRT-PCR Assay

**Figure 3:**
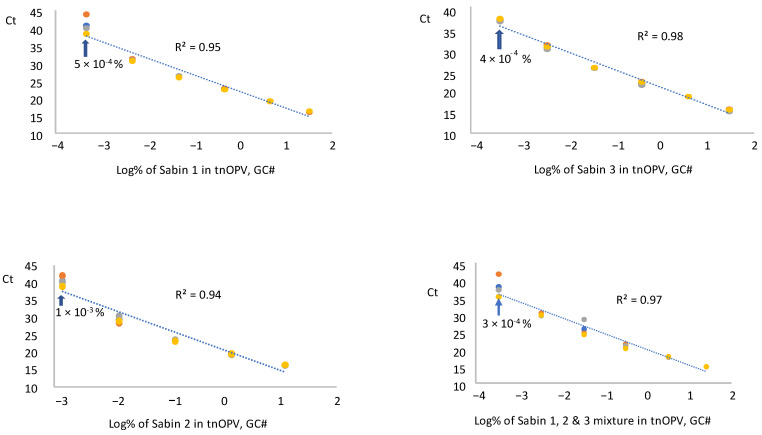
Evaluation of sensitivity and linearity of the panRT-PCR assay (Three repeats were used): Analysis of samples composed of individual Sabin strains and the three Sabin strains mixture spiked in the tOPV. Percentages of Sabin viruses in the tnOPV were calculated based on the titers of the viruses expressed on genome copy number (GC#)/mL.

We tested RNA samples extracted from each Sabin strain and a mixture of the Sabin strains spiked in tnOPV in three replicates by panRT-PCR assay and averaged the resulting Ct values for each replicate. The data were plotted against the respective log_10_ of Sabin percentages (calculated based on virus concentration expressed in GC#/mL), as shown in Figure 3. Results indicated that the assay could detect 5 × 10^−4^%, 1 × 10^−3^%, 4 × 10^−4^%, and 3 × 10^−4^% for Sabin strains 1, 2, 3, and the Sabin mixture, respectively. These low percentages fell within a linear range of 5 logs for the Sabin mixture and Sabin strains 1 and 3, and of 4 logs for Sabin 2, with respective R^2^ values of 0.95, 0.98, 0.97, and 0.94. We obtained similar results when the percentages of Sabin viruses in tnOPV were calculated based on the concentration of the viruses expressed in CCID_50_/mL (Supplementary Figure S1).

To further evaluate the consistency of the panRT-PCR assay in detecting low amounts of Sabin viruses, we analyzed four samples with low percentages of Sabin mixture in tnOPV. Results (Table 3) showed consistent detection of 3 × 10^−4^% (based on GC#) and 4 × 10^−4^% (based on CCID_50_) of the Sabin mixture in tnOPV.

#### 3.3.2. mosRT-PCR Assay

**Figure 4:**
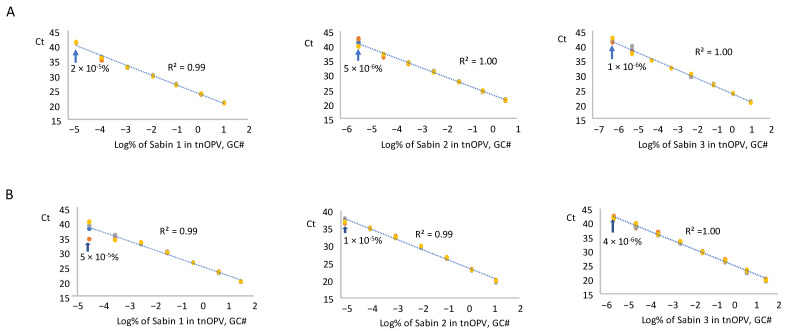
Evaluation of sensitivity and linearity of the mosRT-PCR assay (Three repeats were used): (**A**) analysis of samples composed of individual Sabin strains spiked in the tnOPV; (**B**) analysis of samples composed of the three Sabin strains mixture spiked in the tnOPV. Percentages of Sabin viruses in the tnOPV were calculated based on the titers of the viruses expressed on genome copy number (GC#)/mL.

We tested RNA samples from each Sabin strain and the mixture spiked in tnOPV in three replicates using the mosRT-PCR assay, averaged the resulting Ct values for each replicate, and plotted against the log_10_ of Sabin percentages in tnOPV (calculated based on virus concentration expressed in GC#/mL). Analysis of the Sabin mixture spiked in tnOPV is shown in Figure 4A. The assay detected 2 × 10^−5^%, 5 × 10^−6^%, and 1 × 10^−6^% for Sabin strains 1, 2, and 3, respectively. These values fell within a linearity range of 6 logs for Sabin strains 1 and 2, and 7 logs for Sabin 3, with respective R^2^ values of 0.99, 1.00, and 1.00.

We obtained similar results with the analysis of each Sabin strain spiked individually in tnOPV (Figure 4B). The assay detected 5 × 10^−5^%, 10^−5^%, and 4 × 10^−6^% for Sabin strains 1, 2, and 3, respectively, with a linearity range of 6 logs for strains 1 and 2, and 7 logs for strain 3, and with respective R^2^ values of 0.99, 0.99, and 1.00. We obtained similar results when we calculated the percentages of Sabin viruses in tnOPV based on the concentration of the viruses expressed in CCID_50_/mL (Supplementary Figure S2).

To evaluate the consistency of the mosRT-PCR assay in detecting low percentages of each Sabin strain in the Sabin mixture spiked in tnOPV samples (displayed above in Table 2), we analyzed six samples with low percentages of Sabin 1, 2, and 3 virus mixtures in tnOPV. Results (Table 4) demonstrated consistent detection of 2 × 10^−5^% (based on GC#) of Sabin 1 virus, 5 × 10^−5^% (based on GC#) of Sabin 2 virus, and 1 × 10^−5^% (based on GC#) of Sabin 3 virus.

### 3.4. Analysis of tnOPV Formulated Drug Product Lots (Mock Lots)

We analyzed 12 Formulated tnOPV lots previously evaluated for thermostability [18] using the panRT-PCR assay. Results (Table 5) confirmed the absence of Sabin viruses in the BioFarma tnOPV lots.

We subsequently analyzed the same samples using the mosRT-PCR assay, which corroborated the absence of the Sabin virus in the formulated DP lots (Table 6). The data from both methods showed that the analyzed nOPV strains (negative controls) were negative and that all Sabin strains (positive controls) were positive, confirming the specificity of the methods.

## 4. Discussion

Notable progress has been made in reducing global poliomyelitis cases since the Global Polio Eradication Initiative’s establishment in 1988, which successfully eradicated wild poliovirus types 2 and 3 [3,4]. However, the ongoing circulation of wild poliovirus type 1 in Afghanistan and Pakistan, where 12 cases were reported in 2023 [25] along with persistent outbreaks of cVDPVs [26], complicates the eradication efforts. In recent years, cVDPVs derived predominantly from type 2 Sabin strains have caused more polio cases than wild-type poliovirus, primarily due to outbreaks in Africa, which affected 28 countries in 2023 [27].

The genetically stabilized monovalent nOPV2 vaccine was developed to bolster the ongoing poliovirus eradication efforts [16]. On 23 November 2020, the World Health Organization issued an Emergency Use Listing recommendation for nOPV2 [28,29], enabling its deployment in countries experiencing cVDPV2 outbreaks. Since March 2021, over one billion doses of nOPV2 have been administered in more than 35 countries [30]. Clinical trials and field surveillance data suggest that nOPV2 is both safe and effective and exhibits remarkable genetic stability compared to the Sabin mOPV2 strain [31]. nOPV2 was pre-qualified by WHO in 2023.

Currently, trivalent nOPV (tnOPV), composed of nOPV strains 1, 2, and 3, is being developed at BioFarma [18]. As the production of nOPV vaccine lots progresses, an urgent need exists for simple, sensitive, and specific high-throughput assays to detect potential Sabin poliovirus contamination in nOPV vaccine lots.

Good manufacturing practices and quality assurance are critical for avoiding vaccine contamination and deficiencies. However, contamination can occur at any stage of manufacture, often due to the inadvertent introduction of extraneous agents in raw materials or during manufacturing [32,33,34,35]. The nOPV strains were developed by introducing specific genetic changes to the Sabin 2 genome, enhancing the stability of its attenuation. Given that Sabin viruses can quickly revert to virulence and that nOPV and Sabin OPV vaccines may be processed and/or stocked in the same manufacturing facilities, ensuring batches of nOPV vaccines are free from Sabin virus contamination is essential. Sensitive and specific assays for detecting and identifying Sabin viruses are crucial for quality control and lot release of nOPV vaccines.

Several molecular methods have been developed to detect Sabin strains; however, these methods cannot distinguish Sabin strains from their genetically modified nOPV derivatives, as all primers and probes were designed from the P1 (capsid precursor protein) region of the virus genome common to both nOPV and Sabin OPV viruses [20,21,36,37].

Both PCR and next-generation sequencing (NGS) methods can detect viruses. But PCR-based approaches are generally effective and convenient for analyzing a limited number of targeted samples, especially when the goal is to identify known viruses or when the targeted sample constitutes less than 1% of a mixture, as in the case of vaccine contamination with trace amounts of a virus. In contrast, detecting less than 1% of the targeted sample using NGS is challenging [38] due to the inherent background signal originating from the sequencing process. Furthermore, NGS can achieve decent coverage of the poliovirus genome only when the virus has a titer of at least 10^3^ to 10^4^ CCID_50_/mL. NGS is preferred for high-throughput screening of targeted lots and unknown samples with adequate titers (>10^3^ CCID_50_/mL).

In this report, we described the panRT-PCR and mosRT-PCR assays for the sensitive and specific detection and identification of the three Sabin strains in high quantities of nOPV viruses. The temporary design and acceptance criteria proposed for both panRT-PCR and mosRT-PCR assays aimed to specifically detect Sabin viruses and differentiate them from nOPV viruses. We tested Sabin strain contamination in nOPV RNA samples alongside blank controls (water); nOPV strains 1, 2, and 3 (negative controls); and Sabin strains 1, 2, and 3 (positive controls). All samples were tested in triplicate within the same 96-well plates.

The assay run was considered valid if the following conditions were met at least two of three positive control repeats had Ct values ≤ 45, and at least two of three negative control repeats had undetermined Ct values (or Ct > 45). A test sample was considered positive if at least two of three repeats had Ct values ≤ 45. A threshold Ct of 45 was chosen as a worst-case scenario to maximize sensitivity, even though this threshold might affect specificity under different conditions. Thus, further validation is needed to establish a more definitive Ct threshold to ensure that the assays are specific and function effectively under varying conditions and in different laboratories.

Both assays demonstrated high specificity and sensitivity, with panRT-PCR detecting approximately 10^−4^% of Sabin genomes in tnOPV (which contains more than 10^6^ CCID_50_/mL of each nOPV type). This indicates that the assay can detect one genome copy of Sabin virus amid one million genome copies of nOPV viruses. We observed no cross-amplification with nOPV and Sabin genomes (Figure 2A, Table 5). Additionally, the panRT-PCR assay for detecting Sabin contamination in nOPV stocks exhibited a linearity range of at least 4 log_10_, with an R^2^ value of the dose-response curve of at least 0.94 (Figure 3).

The mosRT-PCR assay detected and identified approximately 10^−5^% of each Sabin strain in tnOPV (which contained more than 10^6^ CCID_50_/mL of each nOPV type), meaning the assay could detect and identify 0.1 genome copy of one Sabin strain among one million genome copies of nOPV viruses. We did not observe cross-amplification between nOPV and Sabin genomes (Figure 2B, Table 6), nor did we note interference among the dyes (FAM, VIC, and NED) used to identify the Sabin strains. Furthermore, the mosRT-PCR assay for detecting Sabin contamination in nOPV stocks displayed a linearity range of at least 6 log_10_, with an R^2^ value of the dose-response curve of at least 0.99 (Figure 4). In both assays, high titers of tnOPV in the tested samples did not affect assay specificity; conversely, sensitivity appeared to improve (Figure 2, Table 3 for panRT-PCR, and Figure 4, Table 4 for mosRT-PCR).

We evaluated these assays using various tnOPV lots prepared from BioFarma bulk. The assays could be used to control the quality of nOPV vaccine lots and detect and identify Sabin viruses as potential contaminants. However, further validation is necessary to account for location, analysts, reagents, and equipment used.

## 5. Conclusions

The panRT-PCR and mosRT-PCR assays for detecting and identifying Sabin virus contamination in nOPV vaccines provide simple and rapid methods for detecting and identifying Sabin viruses, either separately or combined in the presence of nOPV viruses. These assays were specifically designed to ensure quality control during the manufacturing process of nOPV vaccines, effectively identifying Sabin viruses as potential contaminants.

## Figures and Tables

**Table 1:** Primers and TaqMan probes for panRT-PCR and mosRT-PCR assays.

Primer Name (Row Number)	Oligos Sequence 5′ --> 3′	Location	Tm (Basic)	Size (nt)	Amplicon Size (bp)
Primers and TaqMan Probe for Universal Detection of Sabin Viruses in nOPV (panRT-PCR Assay)					
SabUprFv3 (3)	TRGARCAYACCATWAAYAAYTAC	4434–4456 *	45–54	23	70
SabUprRv2 (4)	ACTARCAAACATACTGGYTCA	4503–4483 *	47–48	21	
SabUprbFAM (5)	FAM-ACAGTTCAAGAGCAA-MGBNFQ	4459–4473 *	37	15	
Primers and TaqMan Probe for Detection and Identification of Sabin 1 Virus in nOPV (mosRT-PCR Assay)					
Sab1-670F (7)	GGATTCGCTCCATTGAGTGTGT	670–691 *	55	22	79
Sab1-812R (8)	ACCCATTATGATACAATTGTCTGATTG	748–722 *	54	27	
Sab1PrbVIC (9)	VIC-TAAGTACAATTTCAACAGTT-AMGBNFQ	698–718 *	45	21	
Primers and TaqMan Probe for Detection and Identification of Sabin 2 Virus in nOPV (mosRT-PCR Assay)					
Sab2-538F (11)	CGGAACAGGCGGTCGCGAA	477–495 ^#^	58	19	68
Sab2-605R (12)	GTAGTCGGTTCCGCCACA	544–527 ^#^	57	18	
Sab2PrbFAM (13)	FAM-TGACTGGCTTGTCGT- MGBNFQ	500–514 ^#^	42	15	
Primers and TaqMan Probe for Detection and Identification of Sabin 3 Virus in nOPV (mosRT-PCR Assay)					
Sab3-675F (15)	ATCCACTCCCGAAACGTTTTAC	675–696 ^&^	55	23	75
Sab3-749R (16)	CTCCCATTGTGACACTGAAATCC	749–727 ^&^	55	23	
Sab3PrbNED (17)	NED-TTATTGAAATTGTTTGAAGAC-MGBNFQ	705–725 ^&^	43	21	

Note: Oligos location was mapped on: * Sabin 1 genome (Accession #AY184219); ^#^ Sabin 2 genome (Accession #AY184220); ^&^ Sabin 3 genome (Accession #AY184221).

**Table 2:** Percentages of the spiked Sabin 1, 2, and 3 viruses in the tnOPV.

% of Each of Sabin Strains Spiked in the Mixture of nOPV1, 2, & 3						% of Sabin 1, 2, & 3 Strains Mixture Spiked in the Mixture of nOPV1, 2, & 3							
Sabin 1		Sabin 2		Sabin 3		Sabin 1		Sabin 2		Sabin 3		Sabin 1, 2, & 3	
CCID_50_	GC#	CCID_50_	GC#	CCID_50_	GC#	CCID_50_	GC#	CCID_50_	GC#	CCID_50_	GC#	CCID_50_	GC#
28.41	31.93	4.50	12.37	42.63	29.73	9.48	11.63	1.13	3.50	17.75	10.49	28.35	25.62
3.82	4.48	0.47	1.39	6.92	4.06	1.27	1.51	0.15	0.45	2.38	1.36	3.81	3.33
0.40	0.47	0.05	0.14	0.74	0.42	0.13	0.16	0.02	0.05	0.25	0.14	0.39	0.34
0.04	0.05	5 × 10^−3^	0.01	0.07	0.04	0.01	0.02	2 × 10^−3^	5 × 10^−3^	0.02	0.01	0.04	0.03
4 × 10^−3^	5 × 10^−3^	5 × 10^−4^	1 × 10^−3^	7 × 10^−3^	4 × 10^−3^	1 × 10^−3^	2 × 10^−3^	2 × 10^−4^	5 × 10^−4^	2 × 10^−4^	1 × 10^−3^	4 × 10^−3^	3 × 10^−3^
4 × 10^−4^	5 × 10^−4^	5 × 10^−5^	1 × 10^−4^	7 × 10^−4^	4 × 10^−4^	1 × 10^−4^	2 × 10^−4^	2 × 10^−5^	5 × 10^−5^	2 × 10^−5^	1 × 10^−4^	4 × 10^−4^	3 × 10^−4^
4 × 10^−5^	5 × 10^−5^	5 × 10^−6^	1 × 10^−5^	7 × 10^−5^	4 × 10^−5^	1 × 10^−5^	2 × 10^−5^	2 × 10^−6^	5 × 10^−6^	2 × 10^−6^	1 × 10^−5^	4 × 10^−5^	3 × 10^−5^
4 × 10^−6^	5 × 10^−6^	5 × 10^−7^	1 × 10^−6^	7 × 10^−6^	4 × 10^−6^	1 × 10^−6^	2 × 10^−6^	2 × 10^−7^	5 × 10^−7^	2 × 10^−7^	1 × 10^−6^	4 × 10^−6^	3 × 10^−6^
4 × 10^−7^	5 × 10^−7^	5 × 10^−8^	1 × 10^−7^	7 × 10^−7^	4 × 10^−7^	1 × 10^−7^	2 × 10^−7^	2 × 10^−8^	5 × 10^−8^	2 × 10^−8^	1 × 10^−7^	4 × 10^−7^	3 × 10^−7^
4 × 10^−8^	5 × 10^−8^	5 × 10^−9^	1 × 10^−8^	7 × 10^−8^	4 × 10^−8^	1 × 10^−8^	2 × 10^−8^	2 × 10^−9^	5 × 10^−9^	2 × 10^−9^	1 × 10^−8^	4 × 10^−8^	3 × 10^−8^
4 × 10^−9^	5 × 10^−9^	5 × 10^−10^	1 × 10^−9^	7 × 10^−9^	4 × 10^−9^	1 × 10^−9^	2 × 10^−9^	2 × 10^−10^	5 × 10^−10^	2 × 10^−10^	1 × 10^−9^	4 × 10^−9^	3 × 10^−9^
4 × 10^−10^	5 × 10^−10^	5 × 10^−11^	1 × 10^−10^	7 × 10^−10^	4 × 10^−10^	1 × 10^−10^	2 × 10^−10^	2 × 10^−11^	5 × 10^−11^	2 × 10^−11^	1 × 10^−10^	4 × 10^−10^	3 × 10^−10^

Note: GC#: Percentage calculated with the concentration of the viruses expressed on genome copy number (GC#)/mL. CCID_50_: Percentage calculated with the concentration of the viruses expressed on cell culture infectious dose 50% (CCID_50_)/mL.

**Table 3:** Consistency evaluation of panRT-PCR assay to detect low percentages of Sabin viruses in tnOPV.

% (CCID_50_)	% (GC#)	Run 1 (Ct Repeats)			Run 2 (Ct Repeats)			Run 3 (Ct Repeats)		
		Ct1	Ct2	Ct3	Ct1	Ct2	Ct3	Ct1	Ct2	Ct3
4 × 10^−3^	3 × 10^−3^	29.72	29.05	29.68	30.15	30.34	30.48	30.44	30.14	30.16
4 × 10^−4^	3 × 10^−4^	36.84	34.94	36.02	36.92	37.30	36.64	35.60	35.23	35.30
4 × 10^−5^	3 × 10^−5^	UD	43.18	44.64	UD	UD	UD	UD	UD	UD
4 × 10^−6^	3 × 10^−6^	UD	UD	UD	UD	UD	UD	UD	UD	UD

Note: UD; Not detected.

**Table 4:** Consistency evaluation of mosRT-PCR assay to detect low percentages of each Sabin strain in samples prepared of Sabin strains mixture spiked in the tnOPV.

% (CCID_50_)	% (GC#)	Run 1 (Ct Repeats)			Run 2 (Ct Repeats)			Run 3 (Ct Repeats)		
		Ct1	Ct2	Ct3	Ct1	Ct2	Ct3	Ct1	Ct2	Ct3
Sabin 1 Detection										
0.13	0.16	26.13	25.81	26.07	26.01	26.45	26.14	26.09	25.90	26.00
0.01	0.02	28.93	28.97	28.98	29.18	29.03	29.11	29.02	29.00	28.95
1 × 10^−3^	2 × 10^−3^	31.83	31.33	31.77	31.86	31.95	31.38	31.47	31.77	31.18
1 × 10^−4^	2 × 10^−4^	35.90	34.43	34.66	34.36	34.68	34.50	34.06	33.88	33.93
1 × 10^−5^	2 × 10^−5^	38.27	40.49	37.89	41.36	UD	41.38	41.57	41.29	37.15
1 × 10^−6^	2 × 10^−6^	UD	UD	39.09	UD	UD	43.11	43.06	UD	UD
Sabin 2 Detection										
0.02	0.05	26.93	27.00	26.96	26.50	27.23	27.23	27.51	27.16	26.95
2 × 10^−3^	5 × 10^−3^	30.19	30.06	29.98	30.60	30.60	30.27	30.31	30.33	30.34
2 × 10^−4^	5 × 10^−4^	33.21	32.71	32.93	33.15	33.26	33.12	33.43	33.45	32.98
2 × 10^−5^	5 × 10^−5^	36.71	38.51	35.89	36.19	UD	36.41	35.69	37.14	36.01
2 × 10^−6^	5 × 10^−6^	UD	41.08	UD	41.01	UD	UD	UD	UD	UD
2 × 10^−7^	5 × 10^−7^	UD	UD	UD	UD	UD	UD	UD	UD	35.41
Sabin 3 Detection										
0.25	0.14	25.68	25.61	25.38	25.62	25.89	25.70	25.92	25.90	25.56
0.02	0.01	28.68	28.62	28.25	28.88	28.95	28.62	28.55	28.84	28.37
2 × 10^−3^	1 × 10^−3^	31.09	31.19	30.95	31.27	31.77	31.32	31.10	31.26	30.89
2 × 10^−4^	1 × 10^−4^	34.00	34.59	34.35	34.62	34.60	33.62	33.85	33.44	33.52
2 × 10^−5^	1 × 10^−5^	36.35	36.77	37.71	37.77	37.16	36.16	36.46	36.73	36.14
2 × 10^−6^	1 × 10^−6^	UD	UD	UD	UD	37.93	38.66	38.92	UD	40.69

Note: UD; Not detected.

**Table 5:** Analysis of tnOPV formulation lots with panRT-PCR assay.

tnOPV Lots (Formulation Lots)				Controls	
Sample Name	CT	Sample Name	CT	Sample Name	CT
BF-1	UD	BF-8	UD	Water	UD
BF-1	UD	BF-8	UD	Water	UD
BF-1	UD	BF-8	UD	Water	UD
BF-2	UD	BF-9	UD	nOPV1	UD
BF-2	UD	BF-9	UD	nOPV1	UD
BF-2	UD	BF-9	UD	nOPV1	UD
BF-3	UD	BF-10	UD	nOPV2	UD
BF-3	UD	BF-10	UD	nOPV2	UD
BF-3	UD	BF-10	UD	nOPV2	UD
BF-4	UD	BF-11	UD	nOPV3	UD
BF-4	UD	BF-11	UD	nOPV3	UD
BF-4	UD	BF-11	UD	nOPV3	UD
BF-5	UD	BF-12	UD	Sabin1	16.24
BF-5	UD	BF-12	UD	Sabin1	16.32
BF-5	UD	BF-12	UD	Sabin1	16.48
BF-6	UD			Sabin2	16.56
BF-6	UD			Sabin2	16.61
BF-6	UD			Sabin2	16.72
BF-7	UD			Sabin3	15.29
BF-7	UD			Sabin3	15.11
BF-7	UD			Sabin3	15.46

Note: UD; Not detected.

**Table 6:** Analysis of tnOPV formulation lots with mosRT-PCR assay.

tnOPV Formulation Samples				Control Samples			
tnOPV lot	Sabin1 (CT)	Sabin2 (CT)	Sabin3 (CT)	Sample Name	Sabin1 (CT)	Sabin2 (CT)	Sabin3 (CT)
BF-1	UD	UD	UD	Water	UD	UD	UD
BF-1	UD	UD	UD	Water	UD	UD	UD
BF-1	UD	UD	UD	Water	UD	UD	UD
BF-2	UD	UD	UD	nOPV1	UD	UD	UD
BF-2	UD	UD	UD	nOPV1	UD	UD	UD
BF-2	UD	UD	UD	nOPV1	UD	UD	UD
BF-3	UD	UD	UD	nOPV2	UD	UD	UD
BF-3	UD	UD	UD	nOPV2	UD	UD	UD
BF-3	UD	UD	UD	nOPV2	UD	UD	UD
BF-4	UD	UD	UD	nOPV3	UD	UD	UD
BF-4	UD	UD	UD	nOPV3	UD	UD	UD
BF-4	UD	UD	UD	nOPV3	UD	UD	UD
BF-5	UD	UD	UD	Sabin1	27.42	UD	UD
BF-5	UD	UD	UD	Sabin1	27.80	UD	UD
BF-5	UD	UD	UD	Sabin1	27.47	UD	UD
BF-6	UD	UD	UD	Sabin2	UD	27.64	UD
BF-6	UD	UD	UD	Sabin2	UD	28.16	UD
BF-6	UD	UD	UD	Sabin2	UD	27.84	UD
BF-7	UD	UD	UD	Sabin3	UD	UD	27.42
BF-7	UD	UD	UD	Sabin3	UD	UD	27.70
BF-7	UD	UD	UD	Sabin3	UD	UD	27.82
BF-8	UD	UD	UD				
BF-8	UD	UD	UD				
BF-8	UD	UD	UD				
BF-9	UD	UD	UD				
BF-9	UD	UD	UD				
BF-9	UD	UD	UD				
BF-10	UD	UD	UD				
BF-10	UD	UD	UD				
BF-10	UD	UD	UD				
BF-11	UD	UD	UD				
BF-11	UD	UD	UD				
BF-11	UD	UD	UD				
BF-12	UD	UD	UD				
BF-12	UD	UD	UD				
BF-12	UD	UD	UD				

Note: UD; Not detected.

## Data Availability

All relevant data are within the paper.

## Supplementary Materials

The following supporting information can be downloaded at: https://www.mdpi.com/article/10.3390/vaccines13010075/s1. Figure S1: Evaluation of sensitivity and linearity of the panRT-PCR assay (Three repeats were used). Analysis of samples composed of individual Sabin strains and the three Sabin strains mixture spiked in the tnOPV. Percentages of Sabin viruses in the tnOPV were calculated based on the titers of the viruses expressed in CCID_50_/mL. Figure S2: Evaluation of sensitivity and linearity of the mosRT-PCR assay (Three repeats were used): A, analysis of samples composed of individual Sabin strains spiked in the tnOPV; B, analysis of samples composed of the three Sabin strains mixture spiked in the tnOPV. Percentages of Sabin viruses in the tnOPV were calculated based on the titers of the viruses expressed in CCID_50_/mL.
