# Supplementary material for: Development of RT-PCR Assays for Simple Detection and Identification of Sabin Virus Contaminants in the Novel Oral Poliovirus Vaccines
Source: Vaccines (Basel). 2025 Jan 15;13(1):75. doi: 10.3390/vaccines13010075 (PMC11768561; doi:10.3390/vaccines13010075)
Supplement: Supplementary file 1 [file vaccines-13-00075-s001.zip › Supplemtary Figures S1 and S2.pptx]

## Slide 1
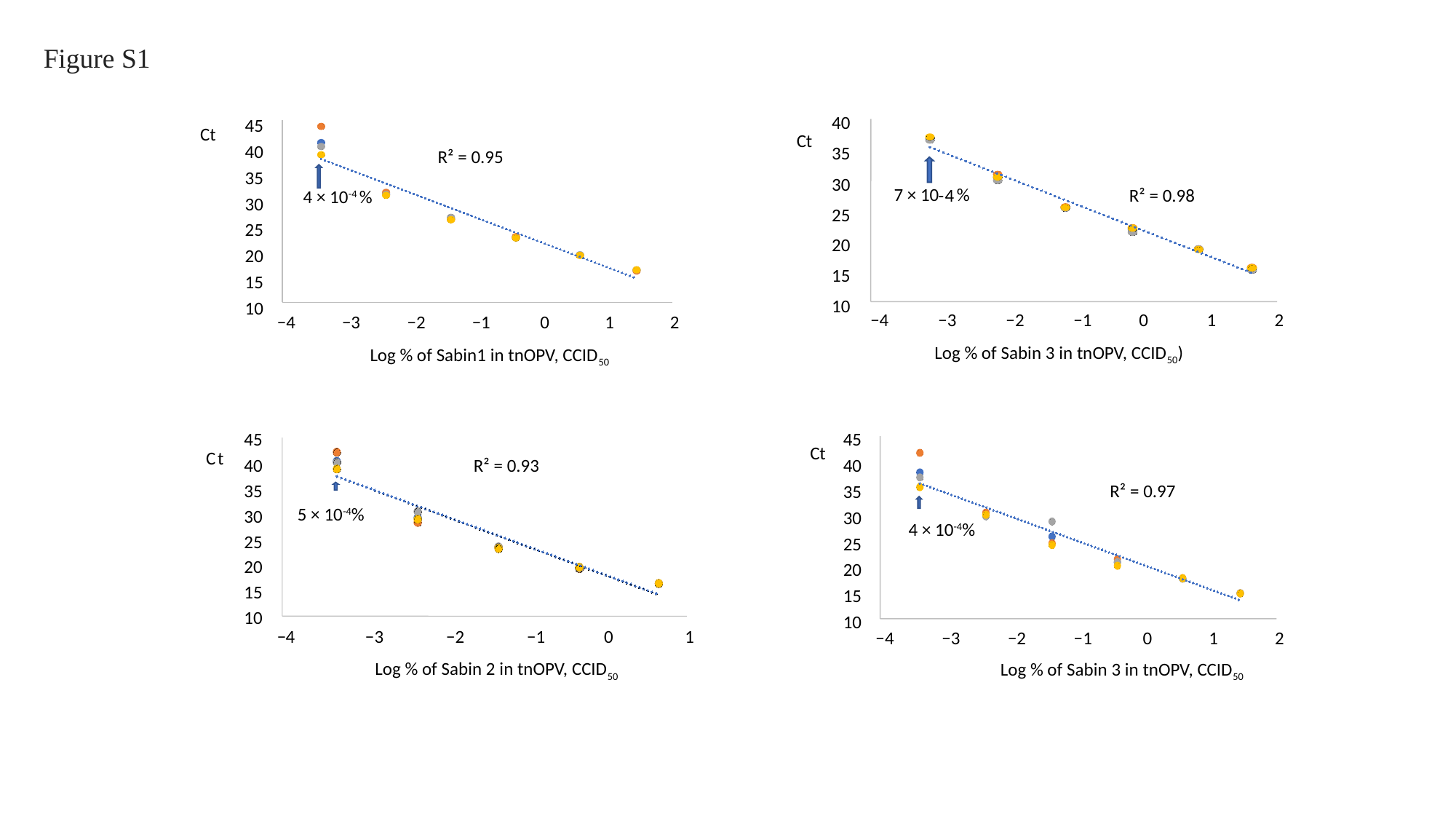

Figure S1
40
Ct
35
30
7 × 10
%
-
4
R² = 0.98
25
20
15
10
2
−4
−3
−2
−1
0
1
Log % of Sabin 3 in tnOPV, CCID50)
45
Ct
40
R² = 0.95
35
4 × 10-4 %
30
25
20
15
10
−4
−3
−2
−1
0
1
2
Log % of Sabin1 in tnOPV, CCID50
45
Ct
40
R² = 0.97
35
30
4 × 10-4%
25
20
15
10
−4
−3
−2
−1
0
1
2
Log % of Sabin 3 in tnOPV, CCID50
45
C
t
40
R² = 0.93
35
5 × 10-4%
30
25
20
15
10
−
4
−3
−2
−1
0
1
Log % of Sabin 2 in tnOPV, CCID50

## Slide 2
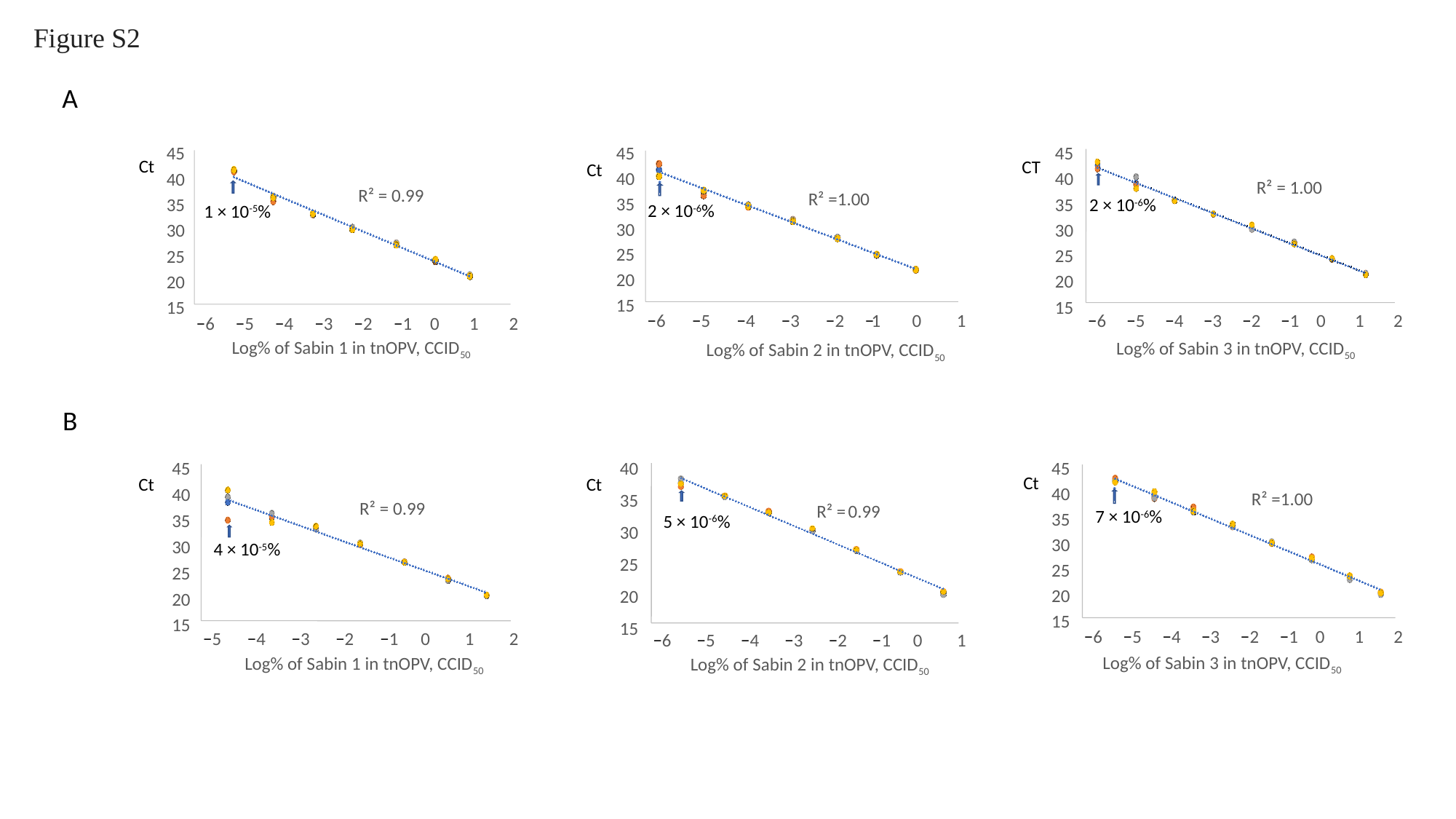

Figure S2
A
45
Ct
40
R² =1.00
35
2 × 10-6%
30
25
20
15
−6
−5
−4
−3
−2
−
1
0
1
Log% of Sabin 2 in tnOPV, CCID50
45
CT
40
R² = 1.00
2 × 10-6%
35
30
25
20
15
−6
−5
−4
−3
−2
−1
0
1
2
Log% of Sabin 3 in tnOPV, CCID50
45
Ct
40
R² = 0.99
35
1 × 10-5%
30
25
20
15
−6
−5
−4
−3
−2
−1
0
1
2
Log% of Sabin 1 in tnOPV, CCID50
B
45
Ct
40
R² = 0.99
35
4 × 10-5%
30
25
20
15
−5
−4
−3
−2
−1
0
1
2
Log% of Sabin 1 in tnOPV, CCID50
40
Ct
35
R² =
0.99
5 × 10-6%
30
25
20
15
−6
−5
−4
−3
−2
−1
0
1
Log% of Sabin 2 in tnOPV, CCID50
45
Ct
40
R² =1.00
7 × 10-6%
35
30
25
20
15
−6
−5
−4
−3
−2
−1
0
1
2
Log% of Sabin 3 in tnOPV, CCID50
